# *Notes from the Field:* Recent Changes in Suicide Rates, by Race and Ethnicity and Age Group — United States, 2021

**DOI:** 10.15585/mmwr.mm7206a4

**Published:** 2023-02-10

**Authors:** Deborah M. Stone, Karin A. Mack, Judith Qualters

**Affiliations:** 1 Division of Injury Prevention, National Center for Injury Prevention and Control, CDC.

Suicide is a serious public health problem in the United States. After 2 consecutive years of declines in suicide (47,511 in 2019 and 45,979 in 2020), 2021 data indicate an increase in suicide to 48,183, nearly returning to the 2018 peak (48,344) with an age-adjusted rate of 14.1 suicides per 100,000 population (versus 14.2 in 2018).* To understand how this increase is distributed across racial and ethnic groups, CDC analyzed changes in racial and ethnic age-adjusted and age-specific suicide rates during 2018–2021.

Suicides were identified from the National Vital Statistics System multiple cause-of-death mortality files for 2018–2021. Age-adjusted rates and 95% CIs were calculated using the direct method and the 2000 U.S. standard population. Hispanic or Latino (Hispanic) persons could be of any race, and racial groups excluded persons of Hispanic ethnicity. Persons with unknown ethnicity were excluded from race and ethnicity groups but were included in the overall total. Differences in rates from 2018 to 2021 were compared using z-tests when deaths were ≥100; p-values <0.05 were considered statistically significant. When deaths were <100, differences in rates were considered significant if CIs based on a gamma distribution did not overlap. This activity was reviewed by CDC and was conducted consistent with applicable federal law and CDC policy.^†^

Age-adjusted 2021 suicide rates were highest among non-Hispanic American Indian or Alaska Native (AI/AN) persons (28.1 per 100,000) overall; this group also experienced the highest relative percentage change during 2018–2021 (from 22.3 to 28.1 per 100,000; a 26% increase) (Table). Age-adjusted rates also increased significantly among non-Hispanic Black or African American (Black) persons (from 7.3 to 8.7; a 19.2% increase) and for Hispanic persons (from 7.4 to 7.9; a 6.8% increase) during 2018–2021. Non-Hispanic White (White) persons were the only group to show an overall age-adjusted rate decline compared with that in 2018 (from 18.1 to 17.4; a 3.9% decline).

**TABLE:** Annual number of suicides and rates of suicide* (suicides per 100,000 population), by race and ethnicity and age group — National Vital Statistics System, United States, 2018–2021

Race and ethnicity^†^/Year	Total no. of deaths	Rate (95% CI)				
		Age-adjusted^§^	Age group, yrs			
			10–24	25–44	45–64	≥65
**American Indian or Alaska Native**						
2018	545	22.3 (20.4–24.2)	31.1 (26.4–35.8)	39.5 (34.7–44.3)	15.8 (12.8–19.4)	—^¶^
2019	546	22.5 (20.5–24.4)	29.9 (25.2–34.5)	39.3 (34.6–44.0)	16.4 (13.3–20.0)	8.0 (5.1–11.8)
2020	588	23.9 (21.9–25.9)	33.0 (28.2–37.9)	41.6 (36.7–46.4)	17.7 (14.3–21.1)	7.2 (4.6–10.9)
2021	692	28.1 (26.0–30.2)	36.3 (31.2–41.3)	52.8 (47.3–58.2)	16.4 (13.3–20.1)	10.7 (7.4–14.8)
Relative rate change, %** 2018–2021	NA	26.0^††^	16.7	33.7^††^	3.8	NA
**Asian**						
2018	1,315	6.7 (6.4–7.1)	8.5 (7.5–9.4)	7.2 (6.6–7.9)	8.2 (7.4–9.1)	8.0 (6.8–9.1)
2019	1,342	6.7 (6.3–7.1)	7.7 (6.8–8.6)	7.8 (7.1–8.5)	8.2 (7.3–9.0)	8.1 (7.0–9.3)
2020	1,302	6.4 (6.1–6.8)	7.4 (6.5–8.3)	7.4 (6.7–8.1)	7.6 (6.8–8.4)	8.3 (7.2–9.4)
2021	1,379	6.8 (6.4–7.1)	9.4 (8.4–10.4)	7.9 (7.2–8.6)	6.9 (6.1–7.6)	7.8 (6.7–8.8)
Relative rate change, %** 2018–2021	NA	1.5	10.6	9.7	–15.9^††^	-2.5
**Black or African American**						
2018	3,022	7.3 (7.0–7.5)	8.2 (7.6–8.8)	11.8 (11.2–12.4)	7.0 (6.5–7.5)	4.4 (3.8–5.0)
2019	3,115	7.5 (7.2–7.7)	8.5 (7.9–9.1)	12.1 (11.4–12.7)	7.1 (6.6–7.7)	4.4 (3.8–4.9)
2020	3,286	7.8 (7.5–8.1)	9.9 (9.3–10.6)	12.7 (12.1–13.3)	6.6 (6.1–7.1)	4.3 (3.7–4.9)
2021	3,692	8.7 (8.4–9.0)	11.2 (10.5–11.9)	14.5 (13.8–15.2)	6.8 (6.3–7.3)	4.4 (3.9–5.0)
Relative rate change, %** 2018–2021	NA	19.2^††^	36.6^††^	22.9^††^	–2.9	0
**Hispanic or Latino**						
2018	4,313	7.4 (7.2–7.7)	7.3 (6.9–7.8)	10.3 (9.8–10.8)	8.6 (8.1–9.1)	7.4 (6.6–8.3)
2019	4,331	7.3 (7.0–7.5)	7.5 (7.1–8.0)	10.3 (9.8–10.7)	8.5 (8.0–9.1)	5.9 (5.2–6.6)
2020	4,571	7.5 (7.3–7.8)	7.9 (7.4–8.3)	11.3 (10.8–11.8)	7.5 (7.1–8.0)	6.9 (6.1–7.6)
2021	4,907	7.9 (7.7–8.1)	7.9 (7.4–8.3)	12.3 (11.8–12.8)	7.8 (7.3–8.3)	6.9 (6.2–7.7)
Relative rate change, %** 2018–2021	NA	6.8^††^	8.2	19.4^††^	–9.3^††^	–6.8
**Native Hawaiian or other Pacific Islander**						
2018	73	11.9 (9.3–15.0)	—^¶^	21.7 (15.6–29.4)	—^¶^	—^¶^
2019	90	14.4 (11.5–17.7)	16.6 (10.3–25.3)	25.9 (19.2–34.2)	—^¶^	—^¶^
2020	79	12.5 (9.9–15.6)	18.9 (12.1–28.1)	18.9 (13.3–26.0)	—^¶^	—^¶^
2021	82	12.6 (10.0–15.7)	16.2 (10.0–24.8)	22.7 (16.6–30.4)	—^¶^	—^¶^
Relative rate change, %** 2018–2021	NA	5.9	NA	4.6	NA	NA
**White**						
2018	38,415	18.1 (17.9–18.3)	12.9 (12.5–13.2)	23.3 (22.9–23.7)	26.1 (25.6–26.5)	20.7 (20.2–21.1)
2019	37,428	17.7 (17.5–17.9)	12.0 (11.6–12.4)	23.0 (22.6–23.5)	25.3 (24.9–25.7)	20.4 (20.0–20.8)
2020	35,442	16.9 (16.7–17.0)	12.0 (11.6–12.3)	22.7 (22.2–23.1)	22.6 (22.2–23.0)	19.7 (19.2–20.1)
2021	36,681	17.4 (17.3–17.6)	12.4 (12.0–12.8)	23.3 (22.9–23.7)	23.1 (22.7–23.5)	20.9 (20.4–21.3)
Relative rate change, %** 2018–2021	NA	–3.9^††^	–3.9	0	–11.5^††^	1.0
**Multiracial**						
2018	514	9.0 (8.1–9.8)	7.2 (6.1–8.3)	13.1 (11.3–14.9)	11.4 (9.2–13.6)	7.2 (4.8–10.3)
2019	527	8.8 (8.0–9.6)	7.2 (6.1–8.3)	13.5 (11.8–15.2)	11.6 (9.4–13.7)	4.7 (2.8–7.2)
2020	599	9.6 (8.7–10.4)	8.0 (6.8–9.1)	15.4 (13.6–17.2)	11.3 (9.2–13.4)	5.7 (3.7–8.4)
2021	631	9.7 (8.9–10.5)	8.2 (7.0–9.3)	15.8 (14.0–17.6)	10.0 (8.1–12.1)	7.4 (5.1–10.2)
Relative rate change, %** 2018–2021	NA	7.8	13.9	20.6^††^	–12.3	2.8
**Total^§§^**						
**2018**	**48,344**	**14.2 (14.1–14.4)**	**10.7 (10.4–10.9)**	**17.9 (17.6–18.1)**	**20.1 (19.8–20.4)**	**17.4 (17.0–17.7)**
**2019**	**47,511**	**13.9 (13.8–14.1)**	**10.2 (10.0–10.5)**	**17.8 (17.5–18.1)**	**19.5 (19.2–19.8)**	**17.0 (16.6–17.3)**
**2020**	**45,979**	**13.5 (13.4–13.6)**	**10.5 (10.2–10.7)**	**17.9 (17.6–18.2)**	**17.4 (17.1–17.7)**	**16.4 (16.1–16.8)**
**2021**	**48,183**	**14.1 (14.0–14.2)**	**11.0 (10.8–11.3)**	**18.8 (18.5–19.1)**	**17.6 (17.3–17.9)**	**17.3 (16.9–17.6)**
Relative rate change, %** 2018–2021	**NA**	**–0.7**	**2.8**	**5.0^††^**	**−12.4^††^**	**−0.6**

**Abbreviation: **NA = not applicable.

* Suicide deaths were identified by using *International Classification of Diseases, Tenth Revision* underlying cause-of-death codes U03, X60–X84, and Y87.0.  † Data for Hispanic or Latino (Hispanic) origin should be interpreted with caution; studies comparing Hispanic origin on death certificates and on U.S. Census Bureau surveys have shown inconsistent reporting on Hispanic ethnicity. Potential racial misclassification might lead to underestimates for certain categories, primarily American Indian, Alaska Native, Asian, and other Pacific Islander decedents. Single-race estimates are presented and might not be comparable to earlier years produced by bridging multiple races to a single race choice. Hispanic ethnicity includes persons of any race. Racial groups exclude persons of Hispanic ethnicity. Persons with unknown ethnicity are excluded from race and ethnicity groups but are included in the overall total.

**^§^** Age-adjusted rates (suicides per 100,000 population) were calculated using the direct method and the 2000 U.S. Census Bureau standard population.

^¶^ Rates are flagged as unreliable or suppressed when the rate is calculated with a numerator of <20, or the data meet the criteria for confidentiality constraints (<10 deaths).

** Relative change was calculated using the following equation: (2021 rate – 2018 rate) / 2018 rate x 100 (for significant difference during 2018–2021, p<0.05. Z-tests were used if the number of deaths was ≥100; nonoverlapping CIs based on the gamma method were used if the number of deaths was ≤100). Data were accessed on CDC WONDER on January 11, 2023.

^††^ Statistically significant at p<0.05.

**^§§^** Includes decedents of unknown ethnicity.

Suicide rates among persons aged 10–24 years increased significantly during 2018–2021 among Black persons (from 8.2 to 11.2; a 36.6% increase). Among those aged 25–44 years, rates increased significantly overall (5%) and among AI/AN (33.7%), Black (22.9%), Hispanic (19.4%), and non-Hispanic multiracial (20.6%) persons during the examined period. Rates among persons aged 45–64 years decreased significantly overall (−12.4%) and among non-Hispanic Asian (Asian) (−15.9%), Hispanic (−9.3%), and White persons (−11.5%). No significant changes were noted among persons aged ≥65 years.

These analyses demonstrate disparities in suicide rates among populations based on race and ethnicity and age group in the context of overall suicide rates nearly returning to their 2018 peak after 2 years of declines. Significant increases among young Black persons aged 10–24 years and across multiple racial and ethnic populations aged 25–44 years raise particular concern. Suicide is a complex problem related to multiple risk factors such as relationship, job or school, and financial problems, as well as mental illness, substance use, social isolation, historical trauma, barriers to health care, and easy access to lethal means of suicide among persons at risk (*1*). Moreover, suicide rates might be stable or even decline during a disaster, only to rise afterwards as the longer-term sequalae ensue for individual persons and within families and communities (*2*). As the nation continues to respond to the short- and long-term impacts of the COVID-19 pandemic, remaining vigilant in prevention efforts is critical, especially among disproportionately affected populations where longer-term impacts might compound preexisting inequities in suicide risk.

The findings in this report are subject to at least three limitations. First, children aged <10 years were excluded from age group category analyses because self-harm intent can be difficult to ascertain in young children (*3*). Second, age-specific rates for some racial groups could not be reported because of small numbers. Finally, racial and ethnic group designation might involve misclassification (*4*).

Research indicates that suicide is preventable through a comprehensive public health approach (*1*) that relies on data to drive decision-making, multisectoral partnerships to expand reach, and implementation and evaluation of multiple culturally relevant prevention strategies. CDC’s Suicide Prevention Resource for Action (*1*) supports states and communities to prioritize interventions with the best available evidence that can save lives. For persons in crisis, help is available through the U.S. Substance Abuse and Mental Health Services Administration’s 988 Suicide & Crisis Lifeline (https://www.988lifeline.org or by texting or calling 988).

## Footnotes

* https://wonder.cdc.gov/mcd-icd10-expanded.html (Accessed January 11, 2023).

† 45 C.F.R. part 46, 21 C.F.R. part 56; 42 U.S.C. Sect. 241(d); 5 U.S.C. Sect. 552a; 44 U.S.C. Sect. 3501 et seq.
